# The Rebuilt Wing at Leeds Infirmary

**Published:** 1911-11-11

**Authors:** 


### The Rebuilt Wing at Leeds Infirmary.

On Tuesday last week the Lord Mayor of Leeds, Mr. William Middlebrook, M.P., reopened the south-east wing of the infirmary, which was partially destroyed by fire last year. The Board, as a result of the fire, decided to make the building fireproof and substituted for the old wood and slate roof one of ferro-concrete, and at the same time accepted plans prepared by their architect, Mr. Sydney D. Kitson, for the entire reorganisation of the block. A full description of the damage caused by the fire appeared in The Hospital of October 15, 1910.

Eight hundred visitors were present at the opening, and Mr. Lupton, who presided, described the wards in detail. The new building, he said, had cost £8,000, £2,800 of which represented insurance money. In referring to the extensions which were made possible by the King Edward Memorial Fund, he paid a tribute to the memory of the late Lord Airedale, who played a great part in the inception of the scheme, and said they were much indebted to the Lord Mayor for what he had done in collecting £110,000, thus making it possible to carry out the modernising of all the wards and the contemplated extensions. The Lord Mayor, in declaring the wards open, said the disastrous fire which necessitated the rebuilding had been fraught with benefit. It had drawn the attention of the Board to the existence of dangers never hitherto suspected. Another advantage was that it provided the opportunity for rebuilding the wing on modern lines. Mr. Middlebrook expressed his thanks for the invitation to retain the presidency of the Memorial Fund, and proposed that the Lord Mayor-elect (Mr. William Nicholson) be treasurer of the fund.

The south-east block, previous to alteration, was detached from the main building and connected only by a wooden bridge and corridor. The staircase, lift, and administration portion of the building was in the centre, thus dividing each of the three floors into two wards, containing respectively ten and sixteen beds. The smaller ward being at the north end was sunless, which, with the drab scheme of decoration, had a depressing effect. This has now been remedied by removing the centre part and placing it at the north end, connecting the block to the main building and making only one long ward containing thirty-four beds on each floor. All the wards open directly on to the principal corridors. The walls are covered to the height of six feet with cream tiles, with the same shade cotinued above in colourwash. The new floors are of three-inch teak boards laid on concrete, the kitchens and lavatory floors being terrazo. The interior of the sanitary towers has been completely removed and replaced by an up-to-date equipment. When the towers were built no allowance was made for cross ventilation between the wards and lavatories; this deficiency has been very ingeniously overcome by the architect, and without the sacrifice of floor space. Over the kitchens and private wards are entresol floors, each containing accommodation for five beds. Although the possibilities of fire are reduced to a minimum a fire-escape staircase has been affixed at the south end and balconies attached for use of patients. The heating and hot-water supply is effected by caloriferes in the basement. In place of the old hydraulic lift has been fixed an automatic electric one. The complete block now accommodates 130 beds in place of the previous 97.

